# Online Health Information and Low-Literacy African Americans

**DOI:** 10.2196/jmir.6.3.e26

**Published:** 2004-09-03

**Authors:** Mehret S Birru, Richard A Steinman

**Affiliations:** ^2^Departments of Medicine and PharmacologyUniversity of Pittsburgh School of MedicinePittsburgh PAUSA; ^1^Department of Basic ResearchUniversity of Pittsburgh Cancer InstitutePittsburgh PAUSA

**Keywords:** Health, literacy, socioeconomic status (SES), African Americans, Internet, comprehension, health behavior

## Abstract

African Americans with low incomes and low literacy levels disproportionately suffer poor health outcomes from many preventable diseases. Low functional literacy and low health literacy impede millions of Americans from successfully accessing health information. These problems are compounded for African Americans by cultural insensitivity in health materials. The Internet could become a useful tool for providing accessible health information to low-literacy and low-income African Americans. Optimal health Web sites should include text written at low reading levels and appropriate cultural references.

More research is needed to determine how African Americans with low literacy skills access, evaluate, prioritize, and value health information on the Internet.

## Introduction

Technologies such as the Internet could conceivably enhance the health knowledge of consumers, but have not adequately reached socioeconomic groups at highest risk for poor health. Disparities in income, education, and treatment account for most of this excess [1], but inaccessible health information also contributes to a higher burden of disease. Many groups encounter obstacles in accessing health information; we concentrate on some of the specific barriers encountered by low socioeconomic status (SES) African Americans with substandard literacy skills. While this group has been shown to suffer excessively from preventable complications of diseases such as breast cancer and diabetes [2,3], minimal research has been conducted to determine the utility of the Internet as a means of improving the accessibility of health information to this population. This paper identifies some of the difficulties related to Web usage many low-SES, low-literacy African Americans, and highlights areas of research that must be examined in order to optimally design resources for this population.

### Background

Mainstream health information is profoundly inaccessible for millions of Americans with low functional literacy. Functional literacy, as defined in the National Literacy Act of 1991, reflects "an individual's ability to read, write, and speak in English and compute and solve problems at levels of proficiency necessary to function on the job and in society" [4]. However, a striking number of Americans are unable to achieve these most fundamental of aims. According to the American Medical Association [5], low literacy limits the ability of 90 million Americans to engage in disease screening or lifestyle modifying activities. People with low literacy are approximately twice as likely to be hospitalized as individuals with high literacy [5], and low literacy has been identified as a barrier to participation in clinical trials [6].

### Our Focus

This paper will focus primarily on defining ways in which informational obstacles-including those found on the Web-may hinder health-seeking behaviors of low-SES and low-literacy African Americans.

While numerous studies have identified low SES as a contributor to the poor health outcomes presented by some African Americans, fewer studies of at-risk African Americans have analyzed their literacy level as another component of their overall health status. However, distinct linkages exist between low literacy, poverty, and poor health.

## Literacy, Income Levels and Health Information

In the 1992 National Assessment of Adult Literacy (NAAL) [7] nearly half of all individuals who read at the lowest defined literacy level (Level 1) also reported the lowest income levels of all participants. Because reports of health complications generally are greatest among low income and undereducated people [7,8], low-SES African Americans are therefore, more likely than high-SES African Americans to present with poorer health.

The NAAL study [7] further reported that 38% of African-American participants were graded at Level 1 for prose literacy, and noted that African American families demonstrate poverty rates up to three times higher than other ethnic groups. The correlation between low literacy and poverty in this group suggests that many studies that describe the health behaviors of low-SES African.

Americans may actually be applicable to behavioral characteristics of African Americans with low functional literacy. Therefore, while there is a paucity of specific research on health-seeking behaviors of low-literacy African Americans, representative results may be found in studies that focus on low-SES African Americans.

However, available research has not clearly correlated functional literacy, SES, and health literacy as contributors to poorer health outcomes in this group. *Health* literacy is defined by researchers at the National Center for the Study of Adult Learning and Literacy as "the ability to use written materials to function in health care settings and to maintain one's health and the skills needed to advocate for and request needed clarification" [9]. Health literacy impacts the health status of some African Americans by hindering their comprehension of health-related topics and their ability to understand health education materials, brochures, and physicians' instructions. A study of African American patients with non-insulin-dependent diabetes in municipal hospital outpatient settings reported that health literacy, as measured by the Test of Functional Health Literacy in Adults, was adequate in only 25% of established patients [10]; this undermined the ability of patients to navigate the health care environment. A study by Morhmann et al [11] determined that "printed educational materials on breast cancer do not adequately provide information to undereducated, economically disadvantaged African-American women", an observation confirmed in other analyses of breast and prostate cancer-prevention materials [12,13].

### Impact on Health Literacy

While the aggregate impact of functional literacy on health literacy has not been clearly identified by research, low functional literacy does limit the ability of individuals to read and comprehend health education materials. Therefore, poor reading skills are likely to limit health literacy and healthy practices supported by written health materials, and may impact health outcomes in the low-SES African-American population [9]. New nationwide initiatives, including the "Ask Me Three" Campaign by the Partnership for Clear Health Communication train the health community to better communicate with low-health literacy individuals; however, inadequacies persist in the development of educationally appropriate materials for African Americans with low incomes and literacy.

### The Internet as an Accessible Health Tool

The Internet may comprise a more accessible, dynamic tool for improving health literacy than current health resources and interventions designed for this group. Internet health Web sites may circumvent some of the typical distribution concerns associated with print health materials. These Web sites may also incorporate multiple mediums to convey information; conceivably this could reinforce comprehension for the low-literacy individuals who access a site.

Is the Internet valued by low-SES African Americans as a source of health information? While no studies directly address this question, Zarcadoolas et al [14] found that health information would be the highest priority search category for low-SES White, Latino/a, and African Americans if they were to access the Internet. Robinson et al [15] further reported that while only 5% of multi-ethnic, low-SES individuals surveyed had used the Internet for health information, nearly half believed that they could find trustworthy and reliable medical information on the Internet . Moreover, home Internet access by low-SES African Americans rose more than three-fold between 1994 and 1998, and African Americans also comprise the largest category of Internet users who access the Web outside of their homes [16].

While these statistics are initially promising, a more comprehensive examination must be conducted to determine the utility of current online health materials for low-literacy, low-SES African Americans, and whether available online resources measurably impact the health literacy of this group. Because no such studies have yet been conducted within this area of research, it is difficult to ascertain whether online health materials are beneficial to this population. However, literacy may be the most daunting barrier to successful Internet access by low-SES, low-literacy African Americans. In one example, researchers noted that 91% of neurology information on the Web was written at a ninth grade or higher level  [17]. Berland et al [18] determined that a collegiate reading level was the average required reading level for 25 English-language health Web sites. The Children's Partnership [19] further indicated that of 1000 Web sites evaluated, only 10 were appropriate for low-literacy adults. These numbers suggest that an alarming paucity of relevant sites exist for all low-literacy individuals. Wilson et al [20] studied ethnic cancer education materials on the National Cancer Institute's CancerNet Web site and demonstrated that the required reading level was 12th grade and cultural references were not adequately specific to the ethnic groups targeted. These results suggest that members of ethnic groups who have low or moderate reading skills may have unique difficulties accessing health information on reputable Web sites.

Health Web sites or pages that are culturally sensitive may, in fact, be particularly important for online African Americans and members of other cultural groups. Culturally sensitive materials present information in a format that reflects the beliefs, practices, and values of a target demographic population. Previous studies have underscored that while racial and ethnic groups consist of highly diverse individuals, visual cues (ie, pictures of African Americans) or lifestyle and historical references may add value to information targeted towards a specific population [21,22]. In one study, Brodie et al [23] sampled opinions of self-reported African Americans towards media health information. Nearly 80% of participants believed that African American individuals and families are visually underrepresented in media health information and 69% believed that inadequate media attention is given to African-American health issues. A 1998 study by Guidry and Fagan [13] supports these perceptions, maintaining that 54% of printed breast- and 40% of prostate-cancer education materials evaluated were not culturally sensitive to African Americans . More research is needed to determine the level of cultural sensitivity of current mainstream online health information resources, and whether it is adequately inviting for usage by low-SES, low-literacy African Americans.

While many mainstream health Web sites (including the American Cancer Society, American Red Cross, and National Institute of Health sites) have attempted to address issues of cultural sensitivity through their development of African-American focused Web pages, hyperlinks to some of these sites are buried and may be difficult to find by an individual with low literacy. For example, the African American pages of the American Diabetes Association are not listed on the homepage and must be accessed through two submenus. Other major disease-specific organizations (eg, cancer, heart disease) lack homepage and submenu linkages to African American-focused Web pages and documents. A mainstream government consumer health search engine, healthfinder.gov, includes hyperlinks to African-American interest sites. Though its hyperlinks to highly sophisticated sites are intact, many hyperlinks to simple health brochures are outdated or unavailable; this may frustrate low-literacy readers who believe their inability to reach selected sites derives from their improper usage of the Internet. In sum, the inclusion of culturally sensitive materials in Web sites may improve user-friendliness for low-literacy, low-SES African Americans, but cannot overshadow the necessity of providing easy-to-read, easy-to-access and easy-to navigate online health materials.

### Overcoming Informational Obstacles on the Internet

Several initiatives seek to exploit the potential of the Internet to empower low-literacy individuals. For example Cyberstep, Inc, a consortium of four literacy organizations operates *thestudyplace.org*, an online educational resource for low-literacy adults. The Adult Literacy Media Alliance (ALMA) produces an online video program (TV411) for low-literacy adults; this addresses some health topics. However, these resources are not specifically developed to address health needs and concerns of poor African Americans.

The full potential of the Internet as a health-promoting medium for low-literacy African Americans cannot be realized with our current state of knowledge. More research needs to be done on the utility of Internet health resources for low-SES and low-literacy African Americans. Several key areas of research would be particularly valuable such as the direct impact of functional literacy on health literacy; search terms and navigation strategies used by low-SES African Americans seeking health information; the concordance between mainstream criteria used in creating/evaluating health Web sites and the criteria used by low-SES African Americans in rating these Web sites [24,25]; and the effectiveness of health information derived from the Internet compared with print medium in enhancing health-promoting behaviors by this population.

It is also critical for research to determine whether the greatest barriers to Internet usage by this group are literacy, socioeconomic status, mechanics related to Internet navigation/usage, cultural considerations, the physical accessibility of Internet resources, and/or other factors currently unidentified. A comprehensive Web site such as the National Cancer Institute's *Usability.gov*, which consolidates research-based conclusions about optimal Web design and usability, may eventually clarify site-design issues and usability needs unique to low-literacy, low-SES African Americans. These findings should eventually inform criteria for health Web site certification by initiatives such as MedCIRCLE [26].

### Design and Content

Ultimately, however, the design and content of health Web sites should be guided by the input of those whom they are meant to serve. A vigorous research agenda is needed to fill in the gaps in our knowledge of how the Internet can best serve the health of low-SES African Americans and others confronting cultural and literacy barriers. Only with feedback from these groups can we understand how, when and why those at greatest risk for disease seek health information on the web, how that information is processed and whether it can help modify lifestyles to promote health. We must also increase our understanding of the criteria by which these groups value information they encounter on the Internet, including the relative value of traditional or alternative health information sources, and the importance of spiritual references, anecdotes or basic biology within health material.

## Conclusion

The Internet offers a mechanism for self-directed health learning with the potential to either broaden health literacy or to spread misinformation. For our patients who bear an excess burden of disease and are willing to learn how to become healthy but are daunted by the complex jargon of medicine, it is time for medicine to speak their language both on the Web and in print.

## References

[1] Grant E N, Lyttle C S, Weiss K B (2000). The relation of socioeconomic factors and racial/ethnic differences in US asthma mortality. Am J Public Health.

[2] Lannin D R, Mathews H F, Mitchell J, Swanson M S, Edwards M S (1998). Influence of socioeconomic and cultural factors on racial differences in late-stage presentation of breast cancer. JAMA.

[3] Klag M J, Whelton P K, Randall B L, Neaton J D, Brancati F L, Stamler J (1997). End-stage renal disease in African-American and white men. 16-year MRFIT findings. JAMA.

[4] Congress n National Literacy Act.

[5] Ad Hoc Committee on Health Literacy for the Council on Scientific Affairs, American Medical Association (1999). Health literacy: report of the Council on Scientific Affairs. JAMA.

[6] Paasche-orlow Michael K, Taylor Holly A, Brancati Frederick L (2003). Readability standards for informed-consent forms as compared with actual readability. N Engl J Med.

[7] Kirsch IS, Jungeblut A, Jenkins L, Kolstad A (1992). Executive Summary of Adult Literacy in America: A First Look at the Results of the National Adult Literacy Survey.

[8] Rudd R, Moeykens BA, Colton TC Vol 1: Health and Literacy. National Center for the Study of Adult Learning and Literacy.

[9] Rudd R, Moeykens BA (1999). Adult educator's perceptions of health issues and topics in adult basic education programs. National Center for the Study of Adult Learning and Literacy.

[10] Nurss J R, El-kebbi I M, Gallina D L, Ziemer D C, Musey V C, Lewis S, Liao Q, Phillips L S (1997). Diabetes in urban African Americans: functional health literacy of municipal hospital outpatients with diabetes. Diabetes Educ.

[11] Mohrmann C C, Coleman E A, Coon S K, Lord J E, Heard J K, Cantrell M J, Burks E C (2000). An analysis of printed breast cancer information for African American women. J Cancer Educ.

[12] Guidry J J, Fagan P (1997). The readability levels of cancer-prevention materials targeting African Americans. J Cancer Educ.

[13] Guidry J J, Fagan P, Walker V (1998). Cultural sensitivity and readability of breast and prostate printed cancer education materials targeting African Americans. J Natl Med Assoc.

[14] Zarcadoolas Christina, Blanco Mercedes, Boyer John F, Pleasant Andrew (2002). Unweaving the Web: an exploratory study of low-literate adults' navigation skills on the World Wide Web. J Health Commun.

[15] Robinson C, Flowers C W, Alperson B L, Norris K C (1999). Internet access and use among disadvantaged inner-city patients. JAMA.

[16] Shapiro R, Rohde G, US National Archives and Records Administration (2000). Falling through the Net : toward digital inclusion : a report on Americans' access to technology tools (SuDoc C 60.2:N 38/2/2000).

[17] Murphy P W, Chesson A L, Berman S A, Arnold C L, Galloway G (2001). Neurology patient education materials: do our educational aids fit our patients' needs?. J Neurosci Nurs.

[18] Berland G K, Elliott M N, Morales L S, Algazy J I, Kravitz R L, Broder M S, Kanouse D E, Muñoz J A, Puyol J A, Lara M, Watkins K E, Yang H, Mcglynn E A (2001). Health information on the Internet: accessibility, quality, and readability in English and Spanish. JAMA.

[19] Lazarus Wendy (2002). Online content for low-income and underserved Americans: The digital divide's new frontier : a strategic audit of activities and opportunities.

[20] Wilson F L, Baker L M, Brown-syed C, Gollop C (2000). An analysis of the readability and cultural sensitivity of information on the National Cancer Institute's Web site: CancerNet. Oncol Nurs Forum.

[21] Guidry J J, Walker V D (1999). Assessing cultural sensitivity in printed cancer materials. Cancer Pract.

[22] Fogel Joshua (2003). Internet use for cancer information among racial/ethnic populations and low literacy groups. Cancer Control.

[23] Brodie M, Kjellson N, Hoff T, Parker M (1999). Perceptions of Latinos, African- Americans, and Whites on media as a health information source. Howard Journal of Communications.

[24] Commission of the European Communities, Brussels (2002). eEurope 2002: Quality Criteria for Health Related Websites. J Med Internet Res.

[25] Eysenbach Gunther, Powell John, Kuss Oliver, Sa Eun-Ryoung (2002). Empirical studies assessing the quality of health information for consumers on the world wide web: a systematic review. JAMA.

[26] Medcircle: The Collaboration for Internet Rating, Certification, Labeling and Evaluation of Health Information.

